# Engram neurons: Encoding, consolidation, retrieval, and forgetting of memory

**DOI:** 10.1038/s41380-023-02137-5

**Published:** 2023-06-28

**Authors:** Axel Guskjolen, Mark S. Cembrowski

**Affiliations:** 1 Department of Cellular and Physiological Sciences, Life Sciences Institute, University of British Columbia, Vancouver, BC, Canada; 2 Djavad Mowafaghian Centre for Brain Health, University of British Columbia, Vancouver, BC, Canada; 3 School of Biomedical Engineering, University of British Columbia, Vancouver, BC, Canada; 4 Department of Mathematics, University of British Columbia, Vancouver, BC, Canada

**Keywords:** Neuroscience, Molecular biology

## Abstract

Tremendous strides have been made in our understanding of the neurobiological substrates of memory – the so-called memory “engram”. Here, we integrate recent progress in the engram field to illustrate how engram neurons transform across the “lifespan” of a memory — from initial memory encoding, to consolidation and retrieval, and ultimately to forgetting. To do so, we first describe how cell-intrinsic properties shape the initial emergence of the engram at memory encoding. Second, we highlight how these encoding neurons preferentially participate in synaptic- and systems-level consolidation of memory. Third, we describe how these changes during encoding and consolidation guide neural reactivation during retrieval, and facilitate memory recall. Fourth, we describe neurobiological mechanisms of forgetting, and how these mechanisms can counteract engram properties established during memory encoding, consolidation, and retrieval. Motivated by recent experimental results across these four sections, we conclude by proposing some conceptual extensions to the traditional view of the engram, including broadening the view of cell-type participation within engrams and across memory stages. In collection, our review synthesizes general principles of the engram across memory stages, and describes future avenues to further understand the dynamic engram.

## Introduction

Memory can be defined as an experience-dependent alteration in behavior that persists beyond the environmental stimuli that produced it. Memory is often conceptualized as a multi-staged process that includes encoding, consolidation, retrieval, and forgetting. As such, mechanistically interpreting memory in the brain is facilitated by understanding the neural underpinnings of each of these stages independently, as well as how these neural elements interrelate across stages. In this regard, significant progress has been made in our understanding memory stages at the level of ‘engram neurons’ – that is, neurons that mediate a particular memory across stages [1–8].

**Fig. 1:**
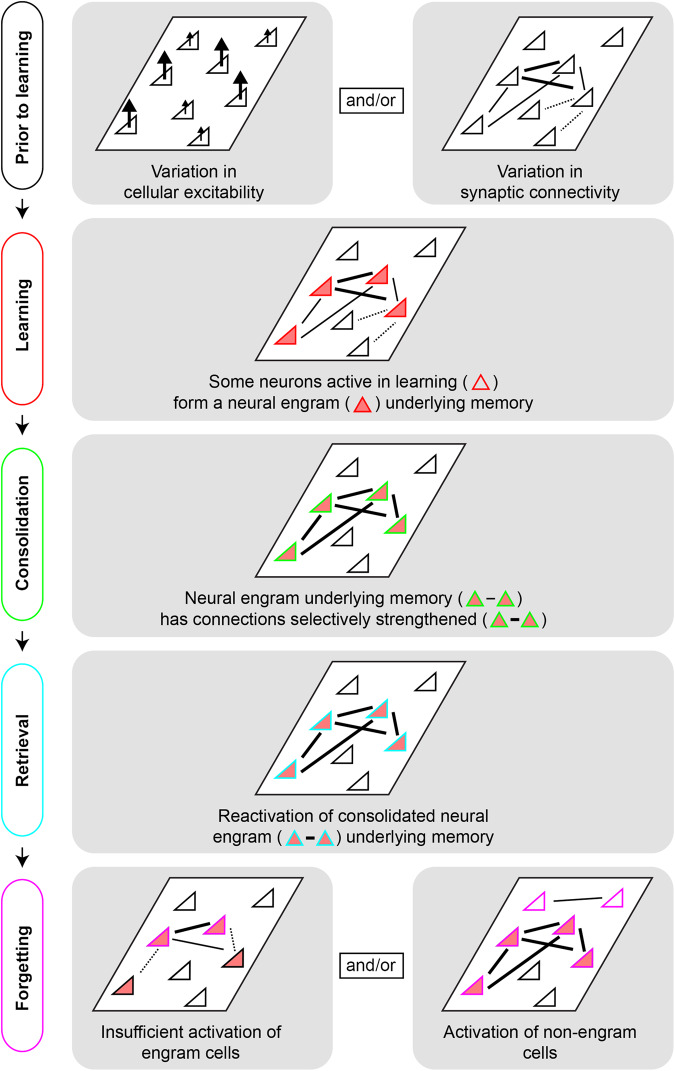
Schematic of cellular and synaptic organization across memory stages. The transformation of the engram is depicted for each stage of memory summarized in this review.

In this review, we seek to identify and connect key overarching principles — principles that seem to largely hold across neural regions and tasks — that lead to neurons participating across multiple memory stages and forming a cellular substrate of memory. Our review focuses primarily on rodent research from the hippocampus, amygdala, and medial prefrontal cortex (mPFC) [9], and is organized according to a typical order of memory stages: encoding, consolidation, retrieval, and forgetting (Fig. 1). We note that many excellent reviews on memory have been written on these memory stages (e.g. [1–3, 5, 8, 10–12]; and note other memory stages exist outside of this scope: e.g. [13–15]). Our goal here is to complement and build upon previous work by synthesizing understanding of engram neurons, both within and across these stages of memory. Motivated by recent empirical developments, we conclude our review by suggesting some important conceptual extensions as to how the engram is traditionally examined and understood.

## Memory encoding

### Intrinsic neuronal excitability regulates recruitment into the engram

The engram can be viewed as the physical change that occurred in the nervous system in response to a learned experience, which can later mediate instantiation of the corresponding memory. As such, engram neurons are typically defined as the neurons that are preferentially involved in the encoding, consolidation, and retrieval of a particular memory [8] (Box 1).

Why are some neurons, rather than their neighbors, recruited during the encoding of a memory? In principle, neurons might be preferentially recruited in memory encoding due to specialized intrinsic properties. Reinforcing this, intrinsic cellular excitability — the propensity of a neuron to fire an action potential in response to input — can be a key determinant of participation in memory [16]. In the context of memory, neurons with high excitability can be biased towards responding during learning and participating in memory encoding [16–18], illustrating in certain cases that intrinsic excitability can bias cells to being active during memory encoding.

To examine this principle from an interventional perspective, the transcription factor cAMP Response Element-Binding protein (CREB) is often leveraged as a tool to regulate neuronal activity during memory encoding [12, 19–21]. CREB regulates neuronal activity in a variety of subcortical and cortical regions [22–26], and across an array of memory tasks [26–30], and can thus regulate which neurons are allocated into the engram. Specifically, CREB-enhanced neurons are preferentially recruited into the engram, whereas CREB-deficient neurons are actively inhibited from encoding the memory [26, 31, 32]. Importantly, exogenous enhancement of CREB minutes before learning is sufficient to promote memory [33], thereby illustrating that CREB activity can shape memory on brief and behaviourally relevant timescales.

Such CREB-dependent recruitment is often posited to reflect changes in intrinsic neural excitability, with these changes in excitability shaping allocation during memory encoding. This posit has been empirically demonstrated in some cases, wherein selective suppression of the excitability of CREB-enhanced neurons prevents their preferential recruitment into the engram, whereas increasing excitability in a subset of neurons without manipulating levels of CREB enhances their recruitment into the engram [34]. This excitability-based mechanism of memory allocation is recapitulated under physiological conditions, with natural fluctuations in rates of neural excitability determining which neurons are selected to encode the corresponding memory [18]. It should be noted that the timescales of excitability plasticity changes can be faster than that of that CREB activity changes, and thus the extent to which natural fluctuations in CREB dictate allocation into the engram has yet to be demonstrated. Finally, it should also be noted that CREB shapes a host of disparate cellular functions, including synaptic plasticity [35]. Thus, CREB-driven neural activity may reflect changes beyond intrinsic properties, and its effects on intrinsic neuronal excitability may only account for some aspects of selection of neurons for memory encoding (Box 2). Ultimately, the extent to which endogenous CREB acts directly on excitability, and the CREB-dependent downstream cellular mechanisms that govern excitability, will be important avenues in future research.

The idea that intrinsic neuronal excitability plays an important role in memory allocation receives additional support from work in place cells [36, 37]. Activating place cells at a specific location promotes the formation of a place field corresponding to that location [38, 39] and targeted activation of place cells drives memory-guided spatial behavior [40], indicating that place cells form an essential neuronal underpinning of spatial memory. Most relevant to the current discussion, only a small subset of neurons become place cells during spatial learning, with most remaining silent [36, 41]. What dictates whether a neuron will become a place cell versus a non-responding silent cell? CA1 neurons that become place cells display higher rates of excitability from the beginning of exploration, and sometimes even before the animal is introduced to the new environment [42]. Remarkably, silent cells can be transformed into place cells with spatially tuned place fields by lowering their activation thresholds [43], potentially suggesting that increased intrinsic excitability may result in place cell emergence under physiological conditions. These data provide complementary CREB-independent support for the idea that the relative excitability of neurons at the time of learning helps determine which neurons will encode the corresponding memory, indicating that this may be a general principle of memory allocation.

In collection, while these findings converge on neural excitability regulating cellular recruitment during memory encoding, they do not negate that other factors influence memory encoding as well. Current evidence illustrates pre-existing patterns of synaptic connectivity (Fig. 1) and synaptic consolidation alongside neuromodulatory factors (Box 2) likely play essential roles here as well. Such mechanisms may emerge from, as well as be complemented by, epigenetic and other cell-intrinsic molecular properties that are engaged during learning and bias neurons towards an enduring role in memory [44–47]. Thus, while work on CREB and neuronal excitability has played (and will continue to play) a foundational role in our understanding of memory allocation, an important avenue for future research is uncovering complementary factors that predispose neurons to be an element of the engram.

Box 1 Engram terminology: What’s in a word? Empirically, cells that comprise an engram typically require two or more of the following properties: (a) being active during initial learning, (b) displaying some persistent physical change in response to learning, (c) reactivating during memory retrieval, (d) being required or sufficient for memory retrieval [1, 2, 8]. Although these criteria provide conditions to constrain and experimentally assess cellular signatures of memory in the brain, it has been noted that such a rigid engram framework may be overly reductionist [200], and that biological memory traces may be less constrained than this framework would imply. In many ways, the engram itself remains a hypothetical biological construct, which must exist but we are only beginning to understand. As a community, it is likely that terms and concepts that we use to understand the relationship between brain and memory will continue to evolve with the acquisition of new experimental tools, data, and analytical methods [201, 202].

Given that engram cells cannot be unambiguously identified prior to retrieval, in our Review we restrict our use of “engram” to conceptual content, as well as discussions of experimental studies that demonstrate the active involvement of neurons in both encoding and retrieval of memory (in line with the general use in the community). In our “Future Considerations” section, we note some potential limitations to current use of the “engram”, and propose some conceptual challenges and extensions to how the engram is typically understood.

Box 2 Intrinsic excitability and neuromodulation: catalysts for synaptic plasticity Changes in intrinsic excitability embody a non-synaptic form of plasticity, which may also influence synaptic plasticity, such that neurons that display high levels of excitability are primed towards later synaptic plasticity [52, 203–205]. For example, neurons that are naturally more excitable at the time of encoding are preferentially allocated, and these same neurons display increased synaptic plasticity during immediate post-learning consolidation. Thus, neural excitability plays a key role in determining whether a given synapse will undergo synaptic consolidation following memory allocation [206]. Consistent with this, suppressing excitability in CREB-enhanced neurons prevents their preferential recruitment into the engram [34], whereas CREBs influence on synaptic plasticity can affect consolidation of the memory without impacting initial learning, per se (indeed, CREB affects LTP but not basal synaptic function) [26, 207]. This line of evidence suggests that neuronal excitability determines which neurons encode a new memory (i.e., memory allocation), whereas synaptic plasticity mediates consolidation of the memory by dictating subsequent synaptic wiring (i.e., synaptic consolidation). Interestingly, CREB-mediated allocation in one region promotes recruitment of synaptically connected partners in other neural regions [208], highlighting the interplay between neural excitability and pre-existing patterns of synaptic connectivity and plasticity that underlies memory encoding (Fig. 1). Thus, CREB plays complementary and dual roles in memory processing, regulating both memory allocation and subsequent consolidation of the memory [19, 21].

Neuromodulators (e.g., acetylcholine, dopamine, noradrenaline and serotonin) can also act as catalysts for synaptic plasticity in the brain [209, 210]. For example, neuromodulation plays a role in subthreshold induction of synaptic plasticity, providing neuromodulators with a gating function that enables targeted strengthening of memory-related synapses. In this way, neuromodulation can interact directly with the excitability of the neuron, providing a mechanism through which targeted synaptic plasticity and memory specificity can be achieved. The extent to which neuromodulatory factors regulate or interact with memory allocation is a developing and exciting avenue for future research.

### Intrinsic neural excitability mediates formation of neuronal ensembles

Thus far, we have discussed the selection process determining which individual neurons are allocated into a memory. However, memory is often thought to be represented not at the level of individual neurons, but at the level of neuronal ensembles – that is, neuronal populations that show consistently synchronized activity in response to a particular stimulus, function, or mental state (e.g., a memory) [48]. Do neuronal ensembles effectively embody independent neurons, or are there specific interrelationships between neurons comprising an ensemble? Two-photon holographic optogenetics has been used to address this question in a highly specific manner [49–51]. Repetitive two-photon optogenetic activation of groups of neurons increases the probability of their firing together *in the absence of external stimulus*, consistent with the formation of a neuronal ensemble [50]. Fascinatingly, these neuronal ensembles are formed via cell-intrinsic upregulation of neural excitability between stimulated neurons, without a concomitant increase in synaptic plasticity (i.e., no new synaptic connections were made between previously unconnected neurons) [51]. These results suggest that memory formation occurs when highly excitable neurons display coordinated activity during memory encoding [4], and that the corresponding neuronal ensemble is formed based on levels of intrinsic excitability rather than alterations in synaptic plasticity per se (Box 2). While future work is needed to clarify these results (e.g., determining the extent to which they generalize across learning conditions), one interpretation is that highly excitable neurons increase the efficiency of already-existing synapses (Box 2). While these findings highlight the role of neuronal excitability in the selection and formation of neuronal ensembles, they do not negate the role of synaptic plasticity in the stabilization or consolidation of these ensembles once they are created. Indeed, in the time following the formation of a neuronal ensemble that underlies a memory, mechanisms of synaptic plasticity would be engaged to consolidate and strengthen this ensemble for future use.

### Encoding summary

A competition-based rule can account for initial memory allocation, wherein excitable neurons and their associated neuronal ensembles out-compete less excitable counterparts for recruitment into the engram. Converging evidence for this rule has been obtained across an array of memory assays and neural regions. This rule therefore seems to be a generalizable feature of learning, and thus key for understanding the mechanisms underlying memory encoding.

## Memory consolidation

### Memory persistence requires synaptic consolidation

Recently encoded memories can be temporarily maintained via learning-induced increased activity [16, 51–55]. However, these memories are labile, highly susceptible to interference, and will rapidly decay without additional maintenance. The transformation of a short-term, labile memory into one that persists long-term requires gene expression and de novo protein synthesis. These processes culminate in increased synaptic coupling between active neurons co-active at the time of learning – a phenomenon called synaptic consolidation [56, 57]. Relatedly, manipulations that disrupt the molecular cascades involved in synaptic consolidation prevent memory consolidation [58, 59]. For example, through its influence on synaptic plasticity, suppressing CREB activity inhibits memory consolidation [57, 60]. Likewise, protein synthesis inhibitors prevent consolidation of memories if administered soon after learning [56, 57]. As such, synaptic consolidation represents a critical point of divergence, wherein consolidated memories survive and have the potential to be retrieved in the future, while those that aren’t targeted for synaptic consolidation may be lost (but see [61]).

### Synaptic consolidation occurs preferentially in neurons active during learning

Neurons preferentially engaged during learning (i.e., putative engram neurons) selectively exhibit hallmark features of synaptic consolidation following memory encoding. For example, transcriptomic analysis has revealed a highly enriched CREB-dependent network that is recruited in engram neurons following contextual fear conditioning [62]. This CREB-dependent transcription promotes structural and functional changes preferentially in engram neurons, and is required for synaptic consolidation [62] (see Box 2). For example, GluR1 AMPA-Rs are preferentially expressed in dendritic spines of active CA1 neurons following contextual fear conditioning [63], and dentate gyrus engram neurons display increased spine density and synaptic strength following contextual fear conditioning [61]. Protein synthesis inhibitors administered immediately after learning abolish these engram-selective changes and culminate in failed memory consolidation [61]. Additionally, synaptic potentiation and the number and size of dendritic spines is selectively increased in engram-to-engram CA3-to-CA1 synapses following formation of a contextual fear memory [64]. Taken together, these results suggest that synaptic consolidation at the molecular, structural, and functional level occur selectively in the neurons active in response to a learning experience.

### Neuronal reactivation drives early consolidation

The probability of forming a long-term hippocampal-dependent memory increases upon repeated behavioural exposures to the learning event, and intriguingly, repeated internal representations of the learning event also occur during behaviourally ‘offline’ periods. During these offline times, such as sleep or quiet wakefulness, patterns of activity among recently active hippocampal neurons is spontaneously replayed. Such replay events occur in either a forward or backward direction [65, 66] in a temporally-compressed format – upwards of 20x faster than occurred during the initial learning experience [67, 68]. Hippocampal replay events occur selectively during sharp wave ripples (a form of high frequency network oscillation) and drive memory consolidation [67, 69–76]. For example, optogenetic increase of sharp wave ripple duration improves consolidation of the corresponding memory [73], and selective disruption of replay prevents consolidation of the corresponding memory [77]. Furthermore, memory replay doesn’t simply reflect the strongest representation rising to the surface, but often occurs for memories most in need of consolidation (i.e., those most at risk degradation) [78, 79]. In accordance with this, targeted reactivation of fear-conditioning-induced lateral amygdala engram neurons during consolidation increases subsequent memory strength [80] (for conceptually similar results in the retrosplenial cortex, see [81]). Moreover, these fear memory engram neurons are preferentially reactivated during sleep, and optogenetically inhibiting their reactivation during sleep (but not later waking periods) prevents memory consolidation [82–84]. These findings converge on the idea that internally generated replay strengthens recently formed memories.

### Hippocampal-dependent memories undergo systems-level consolidation

In the days, weeks, and months (and potentially years, in humans) following synaptic consolidation, the initially hippocampal-dependent component of memory undergoes extreme reorganization and redistribution such that it can be stored and expressed in a hippocampal-independent, mPFC-dependent format. This spatial reorganization of memory is known as systems consolidation. While hippocampal and mPFC-neocortical ensembles representing the same experience can co-exist in the brain [85–87], the phenomenological (or subjective) qualities of the memory depend on which neuronal ensemble is activated. Hippocampal-dependent memories are context-specific and detailed (i.e., episodic), whereas mPFC-dependent memories are associated with a more gist-like quality [87–92]. This is especially true following systems consolidation, after which mPFC ensembles come to represent commonalities among individual experience to generate a more schematized or generalized representation [93–95]. While time since encoding plays an important role in determining whether the hippocampal or neocortical component of the memory will be expressed during memory retrieval (in accordance with standard consolidation theory [96] and results discussed below), other factors such as task demands, attention, and prior knowledge are important factors as well [87].

### Hippocampal engram reactivation drives systems consolidation

**Fig. 2:**
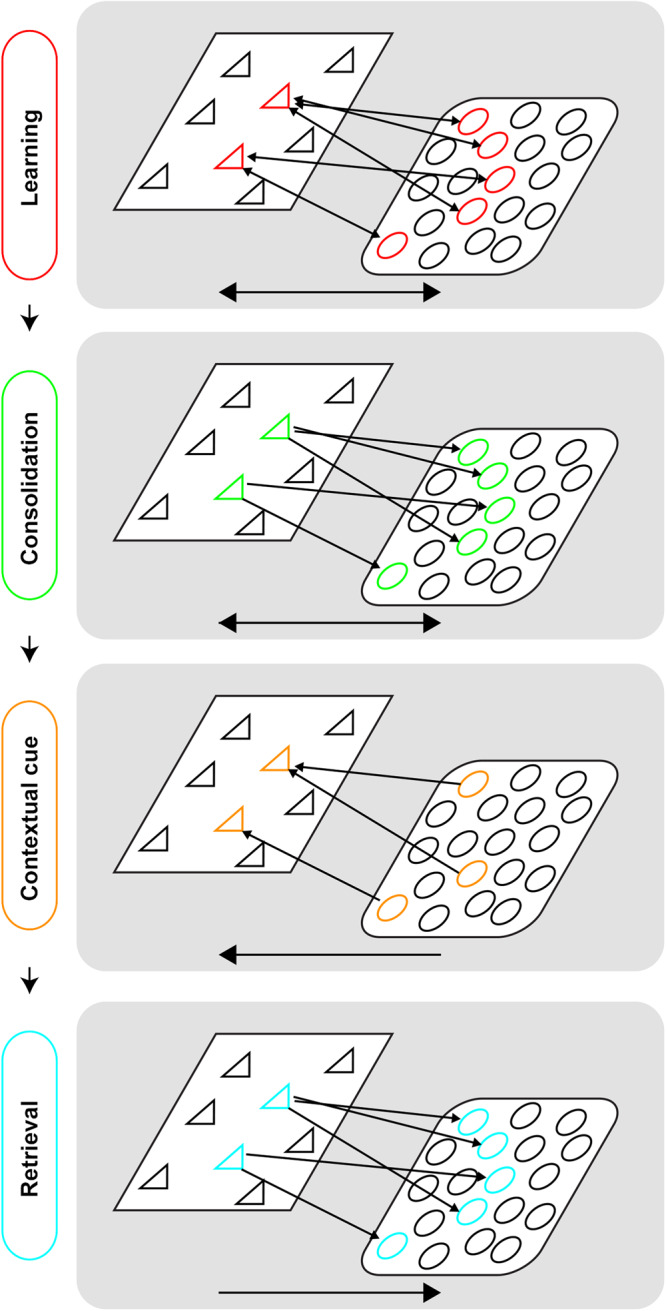
Schematic of indexing theory. Schematic of indexing theory across stages. During initial learning (red stage), communication between an index region (left, akin to the hippocampus) and a perceptual region (right, akin to the neocortex) results in coordinated activity between two ensembles (triangular cells and circular cells, respectively). Consolidation (green stage) strengthens this connectivity between ensembles. Later exposure to a partial contextual cue (gold stage) incompletely activates the perceptual ensemble, but this is sufficient to drive the entire index population. This drives retrieval (teal stage), wherein complete activation of the index cells is sufficient to drive activity across the full perceptual ensemble. At each stage, arrows illustrate flow of information.

In contrast to hippocampal-dependent memories that consolidate within hours, mPFC-dependent memories generally take weeks to consolidate and contribute to memory storage and retrieval [97]. Structurally, mPFC engram neurons take multiple weeks to display learning-mediated increases in dendritic spine density [97], as well as increased synapse-specific strengthening between mPFC engram neurons [98]. What mechanisms promote maturation of mPFC-dependent memory and subsequent completion of systems consolidation? It has long been hypothesized that periods of offline hippocampal activity promote systems consolidation in cortical regions [36, 99]. One parsimonious framework for understanding the role of hippocampus in driving consolidation of a mPFC-dependent memory is indexing theory. According to this idea, the hippocampus forms an index (i.e., a pointer or address book) of the pattern of neocortical activity that was present during initial memory encoding, such that its activation promotes neocortical reinstatement (Fig. 2; Box 3).

One intuitive prediction of the hippocampal indexing theory is that hippocampal activity is required for the maturation and consolidation of mPFC-dependent memory. In line with this prediction, preventing hippocampal engram activity throughout systems consolidation prevents maturation of the mPFC engram, such that these neurons no longer display learning-mediated increases in dendritic spine density, increased engram-to-engram synaptic connectivity, or reactivation during memory retrieval [97, 98, 100] (for conceptually similar results, see [101, 102]). Similarly, place cells in the mPFC require hippocampal activity to form but not to persist [101], and suppressing post-learning hippocampal sharp wave ripples and replay impairs systems memory consolidation [103]. Hippocampal sharp wave ripples become coupled with mPFC spindles (low frequency oscillatory events) following contextual fear conditioning [104], and impairing spindle-ripple coupling post-training prevents consolidation of both recent and remote memory [104]. This result is important, as it suggests that memory replay must be coordinated between the hippocampus and mPFC for memory consolidation to succeed [104]. Reinforcing this, increased hippocampal-mPFC ripple-spindle coupling only occurs when learning results in successful consolidation [105]. Through repeated bouts of hippocampal-to-mPFC replay during sleep, the neocortical memory will stabilize and can eventually be supported independent of the hippocampus, thereby concluding systems consolidation [69–71].

Box 3 Indexing theory: a framework for understanding the role of the hippocampus in memory Indexing theory is a popular framework for understanding the role of the hippocampus in memory processing [211, 212]. According to this idea, the hippocampus forms an index of the pattern of neocortical activity that was present during initial memory encoding (see Fig. 2). Conceiving of hippocampal activity as an index of brain-wide patterns of neural activity that were present during the initial experience provides a parsimonious framework for understanding the role of the hippocampus in both memory consolidation and retrieval. For consolidation, activation of the hippocampal index of a particular memory (e.g., during sleep) would reinstate the corresponding mPFC activity, strengthening the local synaptic connections between these neurons and promote systems consolidation. For retrieval, via the propensity of the hippocampus to reinstate patterns of neocortical activity that were present during the initial experience, the hippocampus helps in promoting recall of detailed episodic memories. Experimental evidence in favor of indexing theory include the finding that inhibiting hippocampal engram neuronal activity suppresses reactivation of cortical engram neurons that were engaged during encoding, and suppresses memory retrieval [97, 98, 100]. Complementary evidence comes from the finding that activating dentate gyrus engram neurons promotes the reactivation of cortical neurons that were active during encoding and retrieval of the corresponding memory [126].

Notably, the hippocampus is not unique in its indexing properties. As one example, the retrosplenial cortex also exhibits indexing-like properties: in the absence of a functioning hippocampus, activation of retrosplenial cortex engram neurons promotes memory retrieval and reinstatement of cortical neurons that were active during encoding [213]. Moreover, ablation of retrosplenial cortex engram neurons suppresses remote memory retrieval [98]. Indeed, it is possible that the mPFC also plays a role in indexing the corresponding neocortical memory, and that this ability becomes more fully realized following systems consolidation. Given that systems-consolidated mPFC activity promotes memories with a more gist-like quality (i.e., lacking the episodic detail of hippocampal-dependent activity), one hypothesis is that the mPFC index is less efficient than the hippocampal index in recapitulating the pattern of neocortical activity that was present during the initial learning experience. In agreement with this, there is dynamic and parallel bidirectional communication between these two regions, with the PFC providing top-down inputs to the hippocampus that are important for memory recall [95].

### Consolidation summary

The transformation of a short-term, labile memory into one that persists long-term requires synaptic and systems consolidation – a process that occurs preferentially in neurons active during memory encoding. Such processes take engram neurons involved in the encoding of memory, and promote connections of both local and long-range ensembles. Such spatially and temporally dynamic processing enables memory persistence via evolving activity that can embody distinct aspects of memory.

## Memory Retrieval

### How we remember: the encoding specificity principle

Once an engram has been consolidated and stored, it can be activated to induce memory retrieval [5]. What dictates successful memory retrieval? According to the *encoding specificity principle*, memory retrieval success is dictated by the extent to which the context (or cues) at retrieval matches that which was present during encoding [106]. The encoding specificity principle can be broken down into the principles of *context-dependent memory* and *state-dependent memory*, which deal with how well the external context and internal neurophysiological state of the animal match between encoding and retrieval, respectfully. The principle of encoding specificity is well established and has been documented in both human and non-human animals using an array of external environments and internal states [107–110].

### Memory retrieval is associated with reactivation of neurons recruited during learning

From the encoding specificity principle, it follows that memory retrieval will be successful to the extent that the brain recapitulates patterns of neural activity that were present during memory encoding. The first evidence of retrieval-induced reinstatement of a putative engram was found by leveraging the time-dependent shift in the location of *Arc* RNA to identify the activation history of individual neurons at two different timepoints (i.e., catFISH). Through this technique, it was found that retrieval of a context memory preferentially reactivates putative CA1 engram neurons that were active during memory encoding [111]. This result was followed up by work using transgenic (TetTag) mice that allowed for the persistent tagging of active (c-Fos^+^) neurons in a narrow time window [112]. Using this system, it was found that fear memory retrieval increased reinstatement of tagged BLA engram neurons, with rates of engram reactivation predicting memory strength [112]. These results provided the first cellular-level observation that memory retrieval is associated with reengagement of the neuronal ensemble that encoded the memory and set the stage for much of what the ‘engram field’ has become today. That memory retrieval is associated with reactivation of neurons that were engaged during learning has been replicated using a variety of engram tagging techniques, in many different memory paradigms, and across an array of neural regions (for review, see [5, 8]).

One inherent challenge of many engram studies is that the experimental context animals are exposed to during memory retrieval is effectively identical to that which they experienced during learning. This experimental design can, in principle, make it difficult to tease apart whether engram reactivation reflects memory retrieval per se, as opposed to these neurons simply becoming active in response to particular features of the environment. Here, it is important to note that natural engram reactivation scales with strength of memory retrieval following fear extinction [13, 98, 112], natural forgetting [113], and in pathophysiological states characterized by memory impairments [13, 114, 115]. That engram reactivation scales with success of memory retrieval provides compelling evidence for a *bone fide* role in memory retrieval, as opposed to simply being evoked by a particular set of stimuli during a memory test.

### Silencing engram neurons disrupts memory retrieval

If the neurons active in response to learning form a critical and enduring component of the memory, then selectively silencing these neurons should disrupt retrieval of the corresponding memory. In a series of influential studies, pre-training neural excitability was amplified in a subset of lateral amygdala or hippocampal neurons, thereby directing a targeted memory into these neurons. Targeted ablation [31] or inhibition [27, 29, 34, 116] of these engram neurons selectively disrupted retrieval of the corresponding memory (without disrupting the ability to learn new information), illustrating that the neural ensemble that encodes memory plays an enduring and necessary role in mediating memory retrieval. Converging evidence for this conclusion have been obtained across an array of neural regions (e.g., dentate gyrus and CA3 [117], CA1 [100], insular cortex [30], nucleus accumbens [118], mPFC [97, 119]) and therefore seems to be a generalizable feature of memory retrieval.

### Activating engram neurons promotes memory retrieval

The encoding specificity principle, and in particular state-dependent memory, suggests that reinstating the state of the brain that was present during memory encoding should promote retrieval of the corresponding memory. While acknowledging the possibility of artificially inducing memory retrieval through direct engram reactivation, most assumed that the spatial-temporal firing patterns underlying memory retrieval were much too precise to be recapitulated via currently available neuron stimulation technology. Indeed, to many (perhaps most) in the field, the possibility of inducing memory retrieval through optogenetic or chemogenetic reactivation of the engram seemed about as likely as recreating Michelangelo’s David with a jackhammer instead of a chisel – our tools were simply too blunt and imprecise. Yet, to the surprise of almost everybody in the field, optogenetically or chemogenetically reactivating ‘memory encoding’ engram neurons induces (partial) retrieval of the corresponding memory, even in the absence of proper environmental retrieval cues [61, 120–122]. Simultaneous activation of engram neurons across multiple brain regions promotes stronger memory retrieval than activation of engram neurons within a single neuron region [86]. That memory retrieval can be induced by activating engram neurons has been widely replicated across neural regions [3, 5, 8] and behavioural paradigms (e.g., fear conditioning [61, 121]; conditioned place avoidance [123] and preference [118], go/no-go licking [49], inhibitory avoidance [114], object location memory [114], social preference memory [124]).

Memory retrieval can promote a transient increase in engram excitability that causally drives improved memory performance [125]. In this work involving contextual discrimination, memory retrieval promotes excitability for hours in dentate gyrus engram neurons. During the period of elevated engram excitability, animals display improved memory flexibility and accuracy in terms of pattern separation and completion at the behavioral level. Mechanistically, this retrieval-induced increase in engram excitability and the corresponding improvement in memory performance is cell-intrinsic, driven by changes in the inwardly rectifying potassium channel Kir_2.1_. Through its comprehensive measurements and direct manipulations of neuronal excitability, this study helps set the standard for casual relationships between engram excitability and memory processing [125].

To uncover robust effects related to memory, most studies in the field seek to manipulate large numbers of engram neurons. In this context, it is striking that optogenetic stimulation of as few as two visual cortex engram neurons is sufficient in driving pattern completion of the neuronal ensemble to which the neurons belong and retrieval of the corresponding memory [49]. Similarly, activation of hippocampal engram neurons promotes the reactivation of (non-stimulated) engram neurons in the amygdala and throughout the cortex [100, 126] (for an indexing theory interpretation of these results, see Box 3). Thus, while the stimulation protocols used in engram activation experiments are focal and largely non-physiological, their effects on the brain are widespread and recapitulate natural patterns of neuronal activity in areas downstream of the stimulated region of interest. The brain’s ability to complete patterns of activity (e.g., brain-wide engram) from incomplete input (e.g., dentate gyrus engram stimulation) likely explains the ability of focal stimulation of engram neurons to drive memory retrieval (Box 4).

Box 4 Limitations of neuronal tagging techniques: What are we missing? The engram field typically labels and/or manipulates neurons that were active during learning. This means that neurons that were not engaged during learning, but which may play a later critical role in regulating memory, are not resolved by current engram labelling techniques. Similarly, neurons whose inhibition (rather than activation) is important in mediating learning and memory are typically not resolved by current engram labelling techniques. Thus, while engram findings illustrate that neurons active during learning play a preferential role in regulating memory, they do not preclude the possibility that neurons active outside of relatively short tagging windows also contribute to memory processing.

Indeed, it seems highly likely that untagged neurons may play important roles in memory processing. From a theoretical perspective, according to multiple trace theory, new memory traces for the same overarching memory can be formed throughout memory consolidation and upon bouts of memory retrieval [214]. Such ensembles would be largely missed through adoption of the experimental design and techniques currently used in the engram field, as they are formed outside of predefined experimental periods (e.g., during a period of offline rest). Completing this theoretical perspective, empirical evidence also illustrates that memory traces can involve non-overlapping populations of neurons in encoding and retrieval [98, 119, 194, 196]). While some of these limitations might be overcome by clever experimental design (e.g., exploiting differential immediate early gene labeling to reveal distinct streams of information [215–217]), other interpretations may be limited by the tag-based approaches used in engram research. Ultimately, the extent to which untagged neurons represent key contributors to memory remains to be seen (see also “Future considerations” section).

### Retrieval summary

According to the encoding specificity principle, memory retrieval success is dictated by the extent to which cues present at retrieval match that those present during encoding. Consistent with this idea, observational experiments illustrate memory retrieval is associated with reactivation of engram neurons engaged during initial learning. Interventionally, silencing neurons that were active during encoding suppresses memory retrieval, whereas activating these neurons promotes memory retrieval. These results hold across an array of tasks and neural regions, suggesting that the engram that is formed during encoding and strengthened during consolidation can be correlatively and causally linked to memory retrieval.

## Forgetting

### Forgetting as an adaptive phenomenon

Forgetting is often viewed as the lack of behavioural expression of a memory, which could be otherwise successfully recalled and expressed on an earlier occasion [11, 127]. According to this perspective, forgetting can occur because the memory is no longer *available* (i.e., complete engram degradation; a storage deficit) or because it is not currently *accessible* (i.e., a retrieval deficit). We note that our operational definition of forgetting here, which can be explained by both storage and retrieval failure, is assayed by behaviour and is agnostic to its biological cause. This varies from some stricter theoretical treatments wherein forgetting requires loss of memory representation per se.

Why do we forget? Memories are perhaps best understood as models of the future [94, 128], and once a memory no longer services predicting what the future might be like, it is best forgotten [129]. While forgetting can occur in a passive manner, for example in response to interfering environmental stimuli, it can also be a well-regulated and active process [130]. Indeed, it has been argued that the natural tendency of neural systems is to degrade rather than preserve information [130, 131]. While forgetting has negative connotations, it is an adaptive phenomenon that promotes future mnemonic processing, decision making, emotional regulation, and mental health [94, 132, 133].

### Synaptic remodeling: A general principle of forgetting

Synaptic plasticity is required for successful learning, but comes at the cost of potentially degrading information already stored in the circuit (i.e. the plasticity-stability dilemma [134]). Given sufficient synaptic remodeling of an engram-specific neuronal ensemble, forgetting is unavoidable: there is an inevitable tipping point beyond which information stored in the ensemble will be lost (i.e., unless another set of synapses takes over the memory representation [135, 136]). This conclusion follows naturally from the encoding specificity principle, and in particular state-dependent memory: as connectivity changes accumulate in an ensemble, the probability of faithfully recapitulating the pattern of activity that underlie memory retrieval within that ensemble diminishes. In this way, synaptic remodeling of engram circuitry represents a general principle of how forgetting occurs in the brain (for review, see [137]).

### Neurogenesis-mediated synaptic remodeling and forgetting

Any significant alteration to the synaptic connectivity within which an engram is embedded should lead to forgetting of the corresponding memory. Post-learning hippocampal neurogenesis is a powerful means through which hippocampal circuitry is remodeled and altered: as new dentate gyrus granule neurons mature, they infiltrate and reconfigure surrounding circuitry by forming connections with both presynaptic and post-synaptic partners [11]. As newborn neurons integrate into these pre-established circuits, their synaptic connections exist alongside and, in some cases, replace established synaptic connections [138–140]. In keeping with their capacity to remodel surrounding neural circuitry, post-learning hippocampal neurogenesis reduces engram reinstatement in downstream CA3 and CA1 [141] and promotes forgetting of the corresponding hippocampal-dependent memory [142–146]. Suppressing the extent to which adult-generated neurons structurally remodel hippocampal circuits (i.e., suppressing their addition of dendritic spines and mossy fiber terminals) prevents neurogenesis-mediated forgetting, whereas increasing the extent to which adult-generated dentate gyrus neurons remodel surrounding circuitry promotes forgetting, even without increasing overall rates of neurogenesis [141]. Thus, neurogenesis promotes forgetting by reconfiguring the circuitry within which hippocampal memories are embedded, thereby decreasing the probability of engram reactivation.

### Microglial and astrocytic regulation of synaptic connectivity and forgetting

Microglia are the brain’s resident macrophage and immune cells. Interestingly, these cells also regulate synapse dynamics and thereby modulate rates of forgetting [147, 148]. Specifically, microglia-mediated synapse removal both decreases dentate gyrus engram reactivation and promotes forgetting of hippocampal-dependent memories [147, 149]. Interestingly, suppressing microglia-mediated synapse elimination also prevents neurogenesis-induced forgetting [147], thereby providing further evidence that neurogenesis induces forgetting via synaptic remodeling of hippocampal circuitry [11]. Astrocytes also regulate synapse dynamics via activity-dependent elimination of excitatory synapses, resulting in forgetting [150]. Interestingly, astrocyte activation can induce NMDA-dependent LTD via postsynaptic GluA2 AMPAR endocytosis [151], suggesting another mechanism through which astrocytes can promote forgetting (see discussion below). Thus, both removal of engram-related synapses [147, 149] and addition of redundant synapses [150] (i.e., bidirectional synaptic remodeling) promotes forgetting of hippocampal-dependent memories.

### NMDA-R and AMPA-R mediated synaptic remodeling and forgetting

Synaptic depotentiation in response to ongoing neural activity (e.g., ordinary mental exertion) is considered one of the main causes of forgetting [152]. Consistent with this, preventing NMDA-mediated synaptic activity (particularly GluN2B-containing NMDA-R activity [153, 154]) blocks synaptic decay and concomitantly prevents forgetting of recently encoded hippocampal memories [153–155]. Similarly, the insertion and stabilization of GluA2-containing AMPA-Rs into post-synaptic sites is associated with synaptic strengthening and memory persistence, and NMDA-dependent removal of these GluA2 AMPA-Rs promotes synaptic depression and forgetting [151, 156–160]. Together, these results suggest that NMDA-R activity and GluA2 AMPA-R endocytosis disassemble the synaptic architecture that was put in place during consolidation; preventing these processes helps preserve the patterns of engram-to-engram synaptic connectivity that underlying the memory, thereby preventing forgetting [130, 159].

### Intracellular signaling: Rac1-mediated regulating of synapse dynamics and forgetting

Intracellular signaling via the Rac1 (a small Rho GTPase) pathway regulates rates of forgetting in flies [161–163], mice [164, 165], and humans [161, 166]. Through its interaction with cofilin (a major promotor of actin cytoskeletal dynamics) [167], Rac1 regulates synapse structure and function and regulates both natural forgetting [164] and forgetting induced by disease states [161]. Whereas decreasing Rac1 activity in the hippocampus prevents synaptic decay and promotes memory persistence, increasing Rac1 availability accelerates synaptic decay and leads to earlier forgetting [164, 165]. Indeed, many of the mechanisms of forgetting already discussed converge on the Rac1 pathway. For example Rac1 activation in dentate gyrus engram neurons promotes microglia-induced synapse elimination and the forgetting of hippocampal-dependent memories [149]. Rac1 also regulates forgetting induced by neurogenesis-mediated circuit remodeling [141] and has been implicated in both NMDAR- and AMPAR-mediated forgetting [168]. Rac1 has also been leveraged to completely remove potentiated engram synapses, resulting in forgetting of the corresponding memory [169]. Together, these results indicate that loss of engram-specific synapses diminishes access to the information stored in that circuit (i.e., forgetting) [170–172] and highlights Rac1 as a key player in this process.

### Neurophysiological noise-induced forgetting: engram reactivation without remembering

Neurophysiological noise that co-occurs during a memory retrieval attempt decreases signal-to-noise ratio and promotes forgetting [173]. For example, altering patterns of synaptic weights via LTP induction in hippocampal synapses promotes forgetting of hippocampal-dependent spatial memories [173]. Moreover, optogenetic or chemogenetic activation of non-engram neurons during memory retrieval promotes forgetting of memories dependent on that circuitry [49, 174–176]. In a somewhat counter-intuitive finding, neurophysiological noise at retrieval often promotes forgetting without decreasing rates of engram neuron reactivation [174, 175](but see [49]). Thus, whereas synaptic remodeling promotes forgetting by decreasing the probability of engram reactivation, neurophysiological noise can interfere with memory retrieval without preventing engram activation. The precise nature of this interference is unknown but likely involves a reduction in memory-related information flow between neural regions. More generally, these results suggest that potentiating synapses that are independent of the engram decreases signal-to-noise ratio, interferes with memory retrieval, and culminates in forgetting.

### Role of engram availability and accessibility in forgetting

The results outlined above suggest that forgetting can occur because the memory is no longer *available* (i.e., engram degradation; a storage deficit) or because it is not currently *accessible* (i.e., a retrieval failure) [177]. Memories are often retrievable in situations where one might classically assume that the engram has degraded to the point where it is no longer available [61]. For example, memories ‘lost’ to infantile amnesia [126, 178] (for related work on infantile amnesia, see [179–181]) and neurogenesis-induced forgetting [141] can be recovered by optogenetic or chemogenetic stimulation of the dentate gyrus engram. Likewise, forgetting in transgenic mouse models of Alzheimer’s disease can be reversed via dentate gyrus engram stimulation [114, 115]. Work that has examined both memory recovery and spine dynamics has found that synaptic strength and spine density can be reduced to baseline levels in hippocampal engram neurons, but nonetheless memory can be recovered via dentate gyrus engram stimulation [61]. Relatedly, selective optogenetic-induced depression of engram synapses induces forgetting, whereas potentiation of these synapses reinstates the memory [170–172, 182].

These results indicate that forgetting is often the result of failed memory retrieval, as opposed to memory erasure. What is the neurobiological explanation for the survival of memory after such drastic synaptic rearrangements and loss of synaptic strength? There are at least three ways of explaining these data. One, some memory-associated synapses remain, and these spared synapses (whether within the targeted neural circuit or in downstream neural regions) are sufficient in storing the memory but not in driving memory retrieval behavior under physiological conditions. Two, the loss of engram-specific synaptic strength diminishes access to information stored in the circuit, but the information stored in the circuit can survive this loss of synaptic strength via persistence in engram-specific synaptic connectivity. According to this explanation, there is a critical distinction between the synaptic strength required for memory retrieval, and the synaptic connectivity required for memory storage (for further discussion and elaboration, see [3, 6, 61, 183, 184]). Three, while highly speculative, it remains possible that non-synaptic mechanisms may be capable of long-term memory storage [3, 47, 185, 186] (Box 5).

Box 5 Non-synaptic mechanisms of long-term memory storage Are patterns of synaptic connectivity the only mechanism of long-term memory storage? The past decade has seen a relative surge in evidence suggestive of non-synaptic mechanisms of memory maintenance. For example, associative fear memories can be reinstated following depotentiation of the synaptic connections generated in response to learning [61, 172], thereby suggesting that memory might be able to persist in a non-synaptic state. Even more striking, simple associative avoidance memories can be transferred horizontally from one animal to another via RNA-induced epigenetic mechanisms in C. elegans [218] and Aplysia [219]. Similarly, simple associative avoidance memories can be transferred transgenerationally via epigenetic mechanisms in C. elegans [220–222], drosophila [223], and possibly even rodents [224, 225]. While epigenetic mechanisms are near the forefront of most theories of non-synaptic long-term memory storage [226, 227], other potential mechanisms include persistent kinase activity [44, 45] and perhaps even via factors outside of cells entirely, such as the patterns of holes in perineuronal nets [228]. Still, we note that it remains difficult to imagine a non-synaptic physiological mechanism through which specific and rapid memory retrieval can occur. For detailed and critical discussions on non-synaptic mechanisms of long-term memory storage, see [47, 183, 186, 229].

### Forgetting summary

As synaptic changes in engram circuitry accumulate, so too does the probability of forgetting. In this way, synaptic remodeling of engram circuitry represents a general mechanism of forgetting. Such synaptic remodeling can occur from a variety of sources, including depotentiation of existing synapses, new synapses driven by ongoing neurogenesis, and synaptic elimination by non-neuronal cells. By disrupting the properties of engram synapses strengthened during early memory stages, circuit remodeling decreases the probability of engram reactivation and promotes forgetting. Nonetheless, engram stimulation experiments can evoke memory retrieval under certain conditions, illustrating that such remodeling does not necessarily produce complete memory erasure per se.

## Future Considerations

### Conceptual underpinnings and extensions of the engram

Engram cells are often conceived as neurons that (a) are active during initial learning, (b) display some persistent physical change in response to learning, and (c) are reactivated during (and required for) memory retrieval [1, 2, 8]. According to this strict definition, no new neurons are added or removed from the engram after it is formed, since these neurons did not participate in both encoding and retrieval (although we note that some definitions incorporate dynamicism; e.g. [7]). Viewed from this perspective, the engram is perceived as a relatively rigid and unchanging neurobiological entity – a fact that seems at odds with the inherently dynamic and constructive nature of memory. Considering this fact, we highlight a few key generalizations regarding the nature of engram cells, motivated by recent experimental progress: (1) non-neuronal engram cells exist, (2) actively inhibited neurons can be an essential component of the engram, (3) different engram neurons can contribute to different stages in memory processing, (4) determining the essential differences (as opposed to only the commonalities) between encoding and retrieval engrams is important to advance the field (i.e., encoding ensemble reactivation is an incomplete model of memory retrieval).

1. ***Existence of non-neuronal engram cells***. Generally, the engram field places a heavy, almost exclusive emphasis on neuronal engram cells. However, it is highly likely that non-neuronal engrams exist, with astrocytes being a prime candidate. For example, emerging evidence suggests that astrocytes play an active role in information process, including regulating synaptic function, circuit connectivity, and memory retrieval [150, 187]. In addition, activation of astrocytes during memory encoding improves memory retrieval without altering basal synaptic transmission [188]. Similarly, the location of a mouse in a familiar maze can be predicted from astrocyte activity alone, suggesting that these cell types might directly encode spatial information [189]. The extent to which astrocytes are instructive engram cells (in addition to being permissive supporting cells) is worthy of serious consideration and experimentation.
2. ***Neural inactivity does not imply mnemonic passivity***. A complete neurobiological understanding of the engram will include not just active neurons, but also actively inhibited neurons. Such active inhibition is often necessary for memory. As one example, the anterodorsal thalamic nucleus is necessary for the retrieval of recent, but not remote, contextual fear memory [190]. Notably, the anterodorsal thalamic nucleus needs to be actively inhibited at remote timepoints for memory retrieval to succeed [190]. Because this inhibition is necessary for content-specific memory retrieval success, these inhibited neurons ought to be considered a genuine component of the engram. Similarly, much as re-activating neurons that were active during encoding promotes memory retrieval, re-inhibiting neurons that were actively inhibited during encoding can also promote memory retrieval [191]. That such neurons are re-inhibited (rather than re-activated) during memory retrieval does not preclude them from being an essential component of the engram (for conceptually related work on inhibitory engrams, see [192, 193]).
3. ***Different neurons often underlie different stages of memory processing***. Engram neurons are typically defined as neurons that were active during both encoding and retrieval of memory. Emerging evidence has illustrated, however, that some neurons play a critical role in memory encoding but do not have a similarly critical role in memory retrieval [194–196]. Conversely, there are neurons that play no clear role in memory encoding, but are recruited into the engram later during consolidation and contribute significantly to memory retrieval [98, 119, 194]. As such, a less strict definition of the engram may serve to help to amalgamate these complementary roles, incorporating ‘*encoding* engram neurons’ (i.e., neurons that are essential for memory encoding only), ‘*retrieval* engram neurons’ (i.e., neurons that are essential for memory retrieval only), and ‘*reactivated* engram neurons’ (i.e., neurons essential to both encoding and retrieval). Such terminology more accurately captures the dynamic nature of memory [7, 197, 198], and better highlights the role different engram neurons play in different stages of memory processing. Conceptually reframing engram neurons in this way could result in new and important research questions. As examples, what are the mechanisms and environmental factors that mediate the recruitment of new neurons into a pre-existing engram? What information is carried by neurons that participate in either encoding or retrieval, but not both?
4. ***‘Encoding’ engram reactivation is an incomplete model of memory retrieval***. Memory is an inherently constructive process. In keeping with this, perception of an experience and memory retrieval of that experience are fundamentally distinct phenomena, with distinct psychological properties, and which must therefore engage – at least in part – distinct neural circuitry [199]. The engram field (including the current article) focuses almost exclusively on the *commonalities* between engram activation at encoding vs retrieval (i.e., engram reactivation) – and this remains a topic worthy of intensive study. However, it is equally important to study and understand the neurobiological *differences* between neural activity during memory encoding vs retrieval. Rather than being interpreted exclusively as noise or mnemonic imprecision, these differences in engram (in)activity could represent important (and adaptive) differences in how the brain processes perceptual information during encoding vs mnemonic information during retrieval.

## Concluding remarks

The engram field, driven by new technology in combination with clever experimental design, has had a truly remarkable rate of recent discovery. The field is now able to visualize, measure, and manipulate engram neurons with an impressive level of specificity, enabling the role and evolution of the engram to be understood across memory stages. Such research has paved the way for exciting future opportunities to understand the engram across memory stages, in both traditional and non-canonical ways, and reveal the logic of memory in the brain.
